# Good Outcome With Respect to Acute Necrotizing Encephalitis in Children Associated With Post-infectious SARS-CoV-2

**DOI:** 10.7759/cureus.43198

**Published:** 2023-08-09

**Authors:** Anayasmin Azmi, Anis Siham Zainal Abidin

**Affiliations:** 1 Paediatrics and Child Health, Medical Faculty, Universiti Teknologi Mara, Sungai Buloh, MYS; 2 Paediatrics and Child Health, Sunway Medical Center, Subang Jaya, MYS

**Keywords:** sars-cov-2, paediatric, tocilizumab, immunotherapy, acute necrotizing encephalitis

## Abstract

This case report demonstrates an excellent outcome in a child with acute necrotizing encephalitis that was likely associated with the post-infectious SARS-CoV-2. The aim of this report is to emphasize that early diagnosis and initiation of immunotherapy treatment may yield good outcome, particularly with the use of tocilizumab and high-dose methylprednisolone. Post immunotherapy, significant neurological improvement was seen through the gradual improvement of Glasgow Coma Scale (GCS) score of 6 to GCS score of 12 within three weeks and subsequently acquired almost full neurological function with minimally dependent activity of daily living (ADL) at eight weeks in the post-treatment follow-up. It is interesting to identify that the possible causative factor could be the natural infection in a vaccinated patient. This was evidenced by the persistently high SARS-CoV-2 immunoglobulin G (IgG)-Spike antibodies titre in a patient vaccinated with two doses of BNT162b2, 11 months before presentation.

## Introduction

Acute necrotizing encephalopathy in children (ANEC) is a known debilitating condition with poor neurological outcomes and high mortality and morbidity rate. ANEC is characterised by viral associated encephalopathy with seizure and rapid drop of Glasgow Coma Scale (GCS), symmetric multifocal brain lesion with bilateral thalami involvement and is often associated with lesion in the cereberal white matter, internal capsule, putamen, brainstem and cerebellum [1]. Further investigations may reveal elevated serum aminotransferases and no CSF (cerebrospinal fluid) pleocytosis [2]. Acute necrotizing encephalopathy scoring system (ANE-ss) may aid to objectively characterise the medium- to long-term outcomes [3]. The higher the score, the poorer the outcome. The landscape of childhood diseases has shifted after the SARS-CoV-2 pandemic. Delayed complications following SARS-CoV-2 infection are seen in children and temporal correlation between COVID-19 infection and increasing number of neurological cases are noted [3]. Testing for SARS-CoV-2 infection and its antibodies in an ANEC patient may lead to some direction for treatment, and immunotherapy could be a part of it.

## Case presentation

We would like to report a single case of an 8-year-old boy, premorbidly well, completed his two doses of BNT162b2 vaccination 11 months before presentation. He presented with short history of fever, vomiting and loose stool one day prior to his presentation to our center, followed by change of behaviour and generalized tonic seizure.

On arrival, he was noted to be encephalopathic with increased tone over all four limbs. His tendon reflexes were increased over bilateral upper and lower limbs with positive babinski and clonus bilaterally. His pupils remained reactive and equal; however, he had rapid deterioration in his hemodynamic status and consciousness level. Prior to intubation, his GCS score was 6, and he was tachycardic with heart rate of 140 beats per minute, hypotensive with the blood pressure of 75/30 mmHg and temperature of 37.8°C. He was intubated for cerebral protection and hemodynamic stabilization.

Reverse transcription polymerase chain reaction (RT-PCR) nasal swab for COVID-19 was negative. PCR nasopharyngeal swab for influenza also was negative.

His blood investigations revealed raised aminotransferase and inflammatory markers. No proof of mycoplasma and human herpes virus 6 (HHV-6) infection were identified. SARS-CoV-2 antibody screening also was sent as part of workout to find the possible causes (Table 1). 

**Table 1:** Relevan investigations results IgM-S: Immunoglobulin M-Spike; IgG-S: Immunoglobulin G-Spike; IgG-N: Immunoglobulin G-nucleocapsid; CSF: Cerebrospinal fluid; PCR: Polymerase chain reaction; HHV-6: Human herpes virus 6

Test	Values	Normal range
RT-PCR COVID-19	Not detected	
PCR nasopharygeal swab for influenza	Not detected	
Aminotransferase	124 U/L	0-31 U/L
White cell count	7 .58	4-11.50 10^9^/L
Platelet	198	150-450 10^9^/L
C-reactive protein	0.6 mg/dL	0-0.5 mg/dL
Erythrocyte sedimentation rate	9 mm/hr	0-15mm/hr
Mycoplasma IgM	Not detected	
HHV-6	Negative	
Interlukin-6	8.11 UL	<3UL
Fibrinogen	222 mg/dL	70 mg/dL
D-Dimer	1.37 ug/mL	<0.5 ug/mL
Ferritin	143.8	7-140 mcg/L
Procalcitonin	9.77 ng/mL	<0.25 ng/mL
SARS-CoV-2 IgM-S	Not detected	
SARS-CoV-2 IgG-S	2311.4 AU/mL	Not detected: <50; Detected: ≥50
SARS-CoV-2 IgG-N	Not detected	
CSF gram stain	Zero cell count, no organism, clear	
CSF chloride	126 mmol/L	<50 mmol/L
CSF protien	410 mg/L	50-400 mg/L
CSF glucose	3.9 mmol/L (ratio 75% of blood glucose)	>50% of blood glucose
Random blood glucose	5.2 mmol/L	3.3-5.8 mmol/L

**Figure 1:**
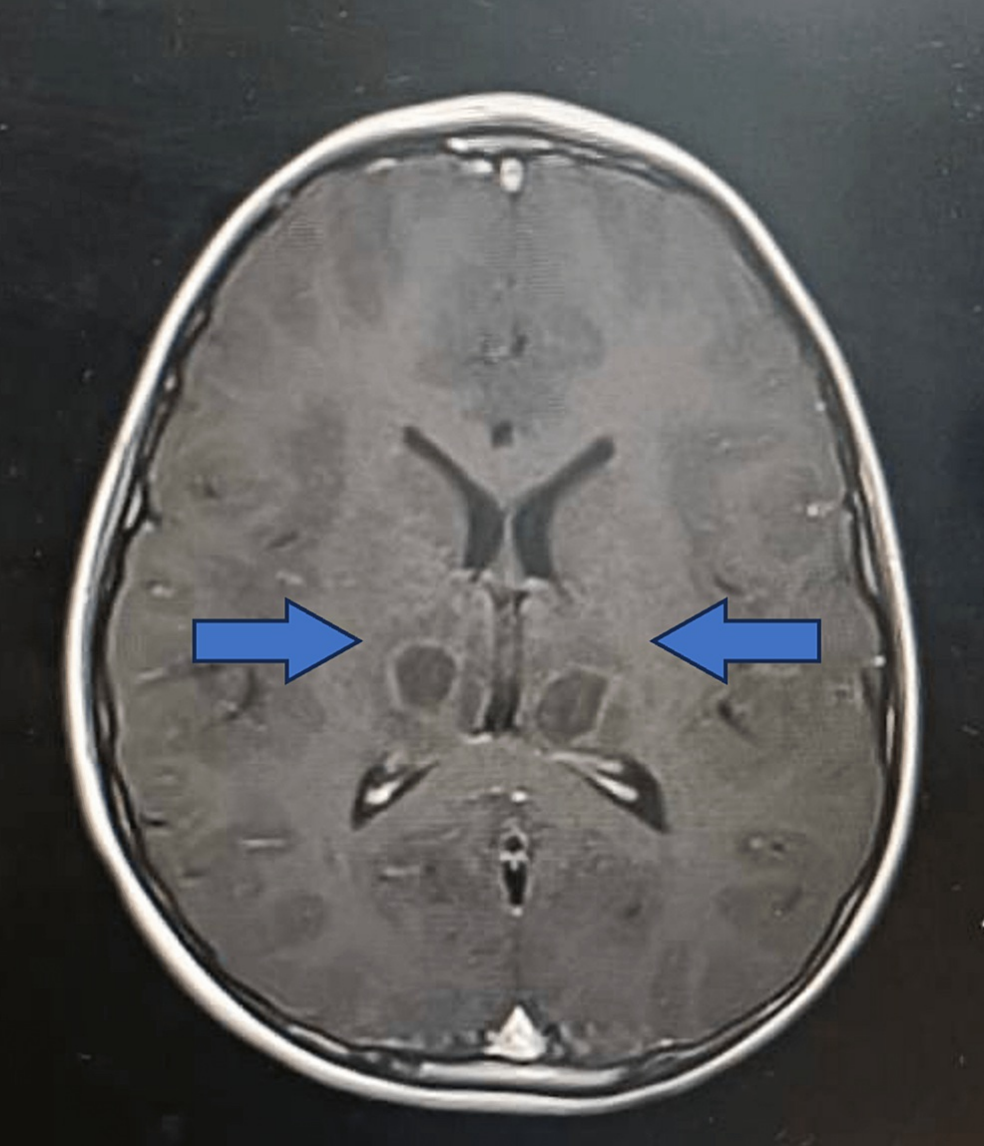
Axial plane MRI shows swelling and symmetrical abnormal signal intensities involving bilateral thalami (arrows).

**Figure 2:**
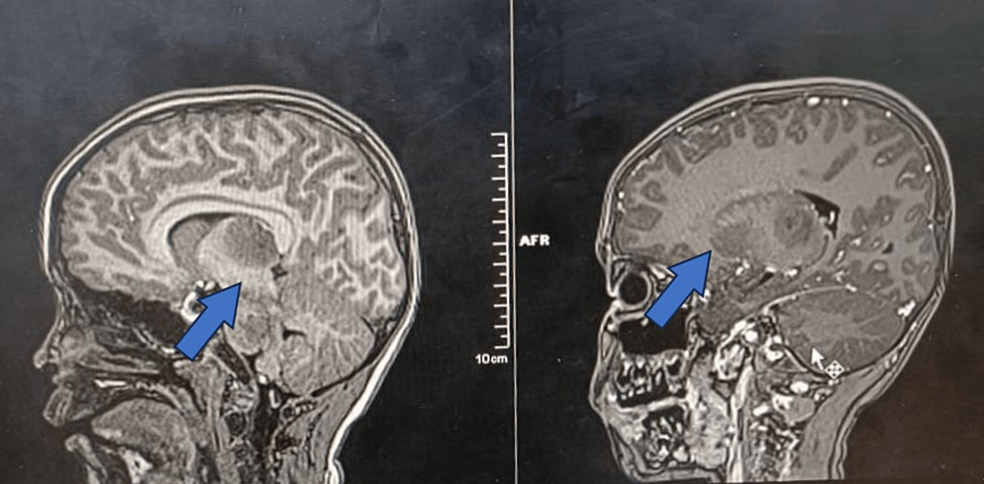
Saggital plane MRI shows swelling and symmetrical abnormal signal intensities involving bilateral thalami (arrows).

Lumbar puncture was also performed. The CSF results noted high in chloride, normal CSF protien and glucose ratio. CSF gram stain showed zero cell count, no organism seen and no CSF pleocytosis. CSF tests were also sent for herpes simplex virus, Japanese encephalitis PCR, anti-N-methyl D-aspartate (NMDA) receptor, and all came back as negative. Echocardiography was performed. No pericardial effusion, coronary arteries were normal, cardiac contractility were good with ejection fraction of 70%. Chest X ray was normal. CT of the brain was performed on the day of presentation showed generalized cerebral oedema with symmetrical thalamic hypodensities with brainstem involvement. MRI of the brain performed on the same day which further confirmed the diagnosis of ANEC (Figures 1,2).

 

Early diagnosis of acute necrotizing encephalitis was made and within 48 hours from symptoms he was immediately started on immunotherapy, which include high dose IV methylprednisolone of 30 mg/kg/dose once daily for five days and single dose of tocilizumab of 12 mg/kg/dose. The prognosis of ANE was measured based on clinical findings by ANE-ss (0-9 point scale). His ANE-ss score of 7 indicated as high risk of poor neurological outcome. The score of 3 points is for shock at onset, 2 points for brainstem lesions, and 2 points for age older than 48 months [4].

## Discussion

In children, SARS-CoV-2 infection is common and frequently asymptomatic. Children may have non-specific symptoms of COVID-19 such as fever and vomiting. The RT-PCR nasal swab to screen for possible parainfectious of COVID-19 was done for this patient and was negative. However, SARS-CoV-2 IgG-Spike antibody test revealed high titre of 2311.4 Au/mL. He completed his second dose of BNT162b2 vaccination 11 months prior to this presentation, which his IgG-Spike antibody was anticipated to be lower. Levin et al. described that humoral response was substantially decreased within six months after the second dose of BNT162b2 vaccination [5]. In another study done by Interiano et al. on paediatric population, they described that the IgG levels in children aged 0 to 11 years old is significantly reduced since day 121 post vaccination. [6]. The persistent high titre of SARS-CoV-2 IgG-Spike antibody in this patient may be due to post-recent infection of SARS-CoV-2, which may be associated to the ANE. This is in line with the findings by Małgorzata Łysek-Gładysińska et al., which mentioned that infection promotes longer maintainance of SARS-CoV-2 antibodies after the second dose of vaccination againts COVID-19. The etiology and pathogenesis of ANEC remains unknown. Causative agents that have been reported include influenza A virus, mycoplasma, herpes simplex virus, and HHV-6, which were all tested negative for this patient [7]. This became important as ANEC is now believed to be most likely immune-mediated or metabolic. It has been reported that cytokines, such as tumor necrosis factor receptor-1, interleukin-1 (IL-1), and IL-6 could mediate the disease [8,9]. Kaye and Siegel highlighted that IL-6 inhibitor tocilizumab could potentially suppress the effects of pro-inflammatory cytokine [10]. The use of tocilizumab as an IL-6-receptor inhibitor in early phase may benefit a patient with ANEC. ANE-ss scoring is to measure the prognosis of ANE. One of the points included in ANE-ss scoring is the brainstem lesions. With score of 7 for his ANE-ss, this patient was expected to have poor prognosis in term of his neurological outcome.. However, we have come to realize that initially, after extubation, his GCS remained poor with the score of 6. He then gradually showed improvement in his neurological functioning to GCS score of 12 within three weeks after initiating treatment, and a later follow-up at eight weeks revealed that his neurological outcome had improved tremendously with full GCS and minimal dependency of his ADL, where he could eat as normal child, talk normally, and started to walk with support. This possibility related to the earlier controlled of the inflammatory storm that occurred in ANEC.

## Conclusions

ANEC is often associated with poor prognosis especially with brainstem involvement. However, early diagnosis and treatment with immunotherapy may lead to good neurological outcome. Post-infectious SARS-CoV2 may be a potential cause.
